# Supplementary material for: Stroke induces disease-specific myeloid cells in the brain parenchyma and pia
Source: Nat Commun. 2022 Feb 17;13:945. doi: 10.1038/s41467-022-28593-1 (PMC8854573; doi:10.1038/s41467-022-28593-1)
Supplement: Supplementary file 1 — Supplementary Information [file 41467_2022_28593_MOESM1_ESM.pdf]

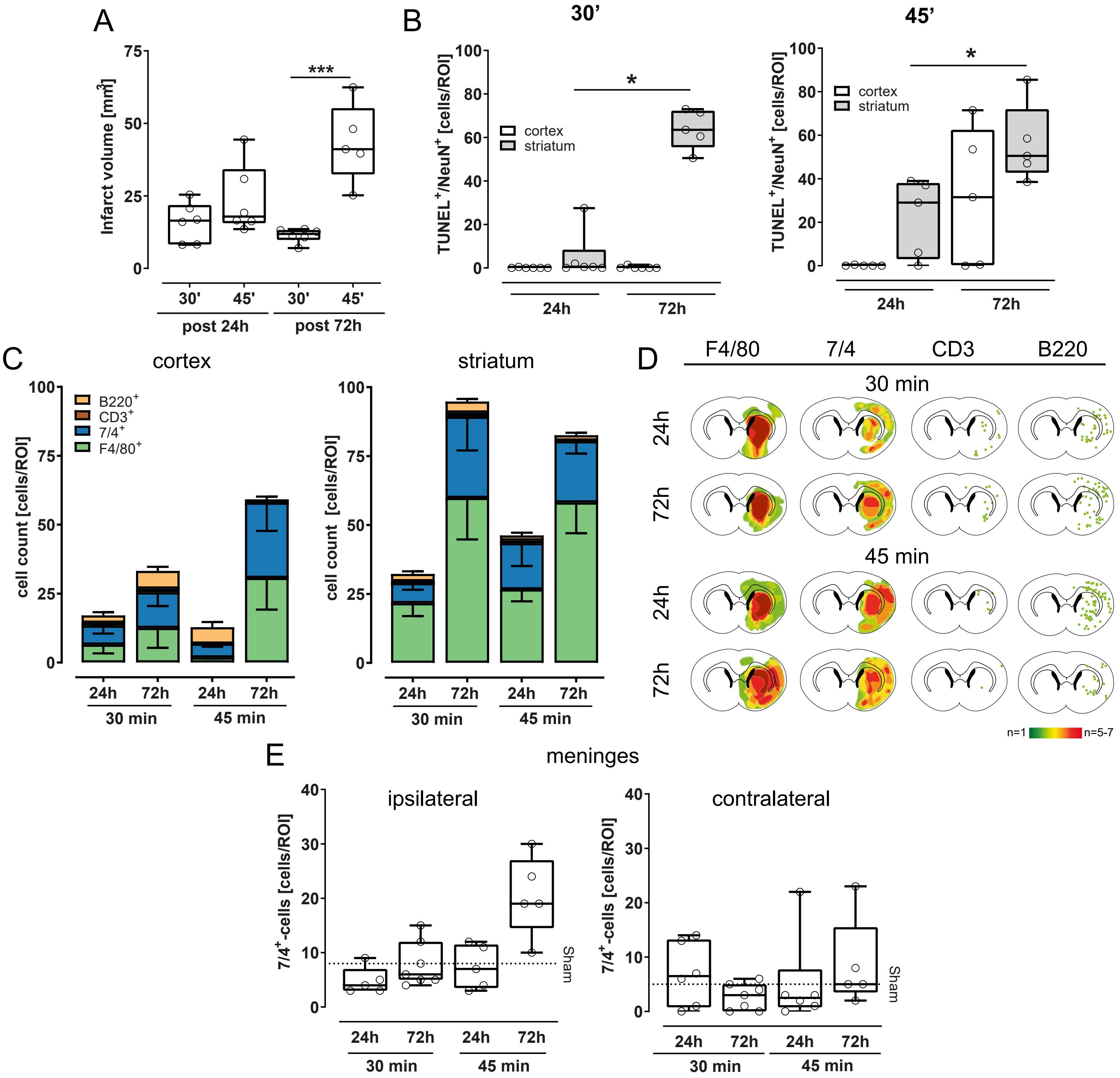

Supplementary Figure 1: MCAO induces ischemia duration-dependent immune cell infiltration and neuronal loss. **(A)** Middle cerebral artery occlusion (MCAO) was induced in mice for 30 or 45 min (each n=5). Mean infarct volumes were calculated from coronal cryosections (15-20) collected at 300 µm intervals and stained with toluidine blue using ImageJ software at 24h and 72h after MCAO (two-sided t-test, ***p<0.001). Median infarct volumes were presented as box plots. Lines inside the boxes denote the medians. Whiskers are: 10–90%. **(B)** TUNEL/NeuN double-staining was performed on cryosections at bregma 0-1 mm. Cells were quantified within the region of interest (ROI: striatum or cortex of ischemic hemisphere, measuring 0.256 mm^2) on coronal sections at 20x magnification. Data are from 4-6 mice and are presented as mean ± SEM in panels A and B. Median cell counts are presented as box plots. Lines inside the boxes denote the medians. Whiskers are: 10–90%. **(C)** Quantification of F4/80$^+$, 7/4$^+$, CD3$^+$ and B220$^+$-cells at 24 and 72h post stroke induction. Cells were quantified within the region of interest (ROI: striatum or cortex of ischemic hemisphere, measuring 0.256 mm^2) on coronal sections at 20x magnification. Data summed from 5-7 mice and are presented as mean ± SEM (two-sided t-test, *p<0.05). **(D)** Heat map (drawn manually using Adobe Illustrator; Methods) depicting the distribution of F4/80$^+$, 7/4$^+$, CD3$^+$ and B220$^+$-cells quantified by immunohistochemistry after 24h and 72h of 30/45 min of MCAO with color legend indicating the quantitative distribution of immune cells. **(E)** Quantification of 7/4$^+$ cells during 24-72h post stroke induction within the ipsilateral (ischemic hemisphere) compared to the contralateral (non-ischemic hemisphere) meninges. Region of interest (ROI) refers to the meninges (pia and dura) found on each cryosection. Data from 5-7 mice and are presented as mean ± SEM. Data of sham-operated wild type mice (n=5) that served as controls are shown with dotted lines. Median cell counts were presented as box plots. The lines inside the boxes denote the medians. The whiskers of box plots: 10–90%. Source data for A, B, C and E are provided as a Source Data file.

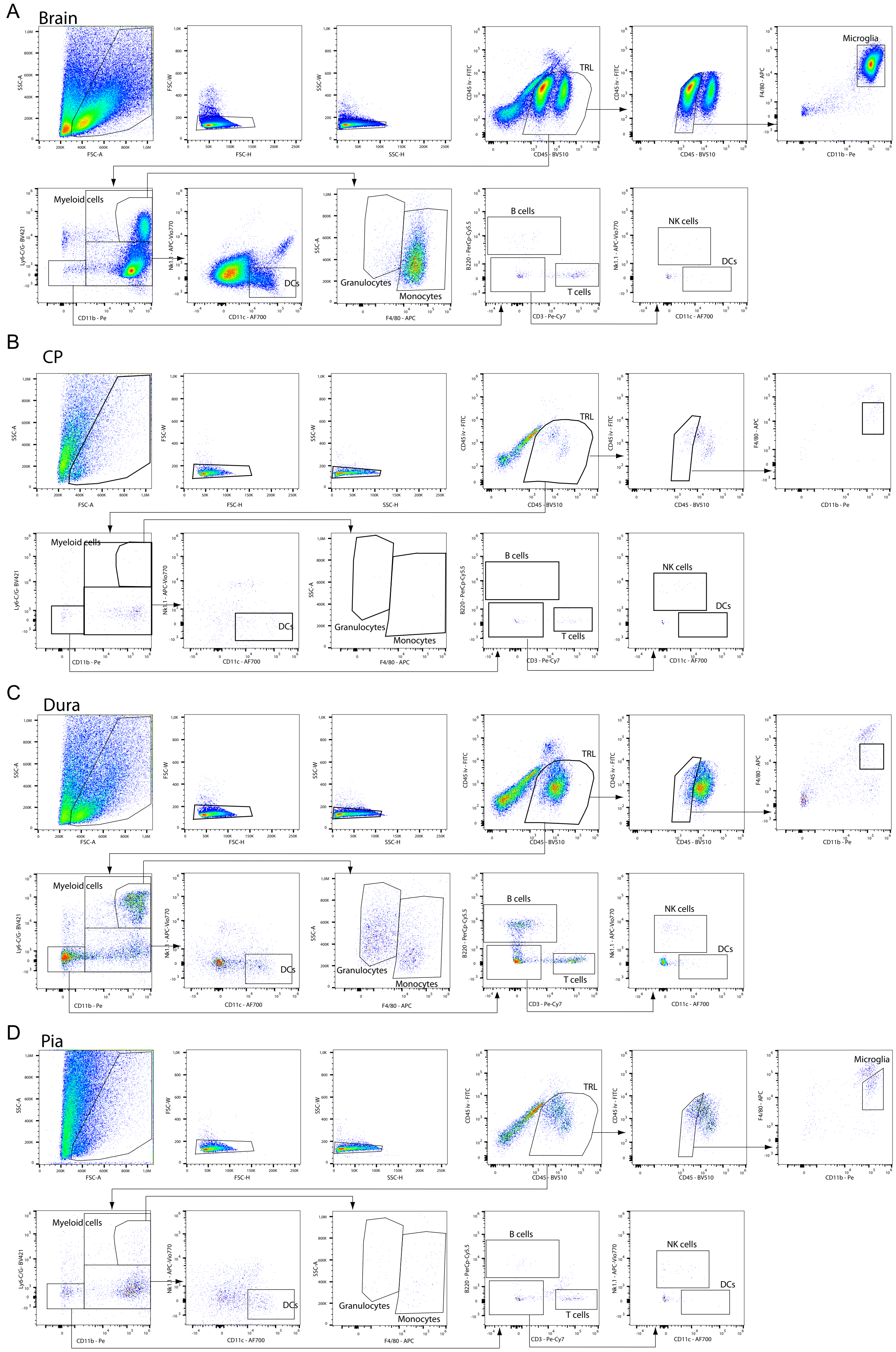

Supplementary Figure 2: Gating strategy to identify cell types by flow cytometry. **(A)** Representative gating strategy of the main cell types identified by flow cytometry in the Brain of 72h MCAO mice as depicted in Fig. 1 B. **(B)** Representative gating strategy of the main cell types identified by flow cytometry in the choroid plexus (CP) of 72h MCAO mice as depicted in Fig. 1 B. **(C)** Representative gating strategy of the main cell types identified by flow cytometry in the Dura of 72h MCAO mice as depicted in Fig. 1 B. **(D)** Representative gating strategy of the main cell types identified by flow cytometry in the Pia of 72h MCAO mice as depicted in Fig. 1 B. TRL, tissue resident leukocytes ($CD45iv^{-}CD45^{+}$); DC, Dendritic cells; Granulo, Granulocytes; Mono/Macro, Monocytes/Macrophages; Bc, B cells; Tc, T cells; NK, Natural killer cells.

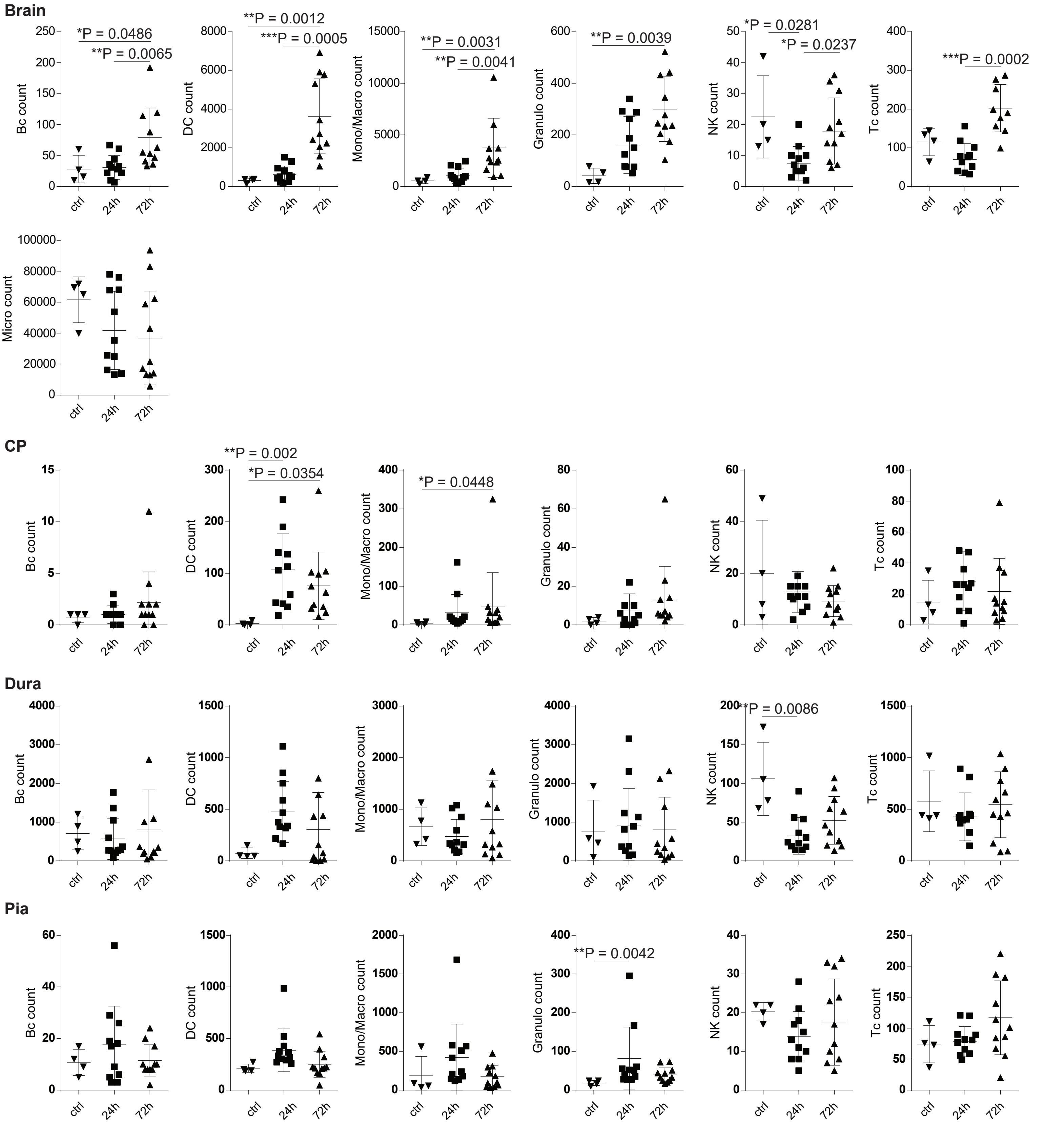

Supplementary Figure 3: Absolute cell counts per mouse identified by flow cytometry. Total cell counts of Bc (B cells), DC (dendritic cells), Mono/Macro (Monocytes/Macrophages), Granulo (Granulocytes), NK (Natural killer cells), Tc (T cells) and Micro (Microglia) of tissue-resident leukocytes isolated out of the Brain, choroid plexus (CP), Dura and Pia from sham-operated mice without stroke (ctrl), from mice 24h post ischemia (24h) and from mice 72h post ischemia (72h) analyzed by Flow Cytometry. n = 4 for ctrl, n = 12 for 24h and 72h. Statistical significance was tested using Kruskal-Wallis test with Dunn's post test. not significant = not shown, *P≤0.05, **P≤0.01, ***P≤0.001. Source data are provided as a Source Data file.

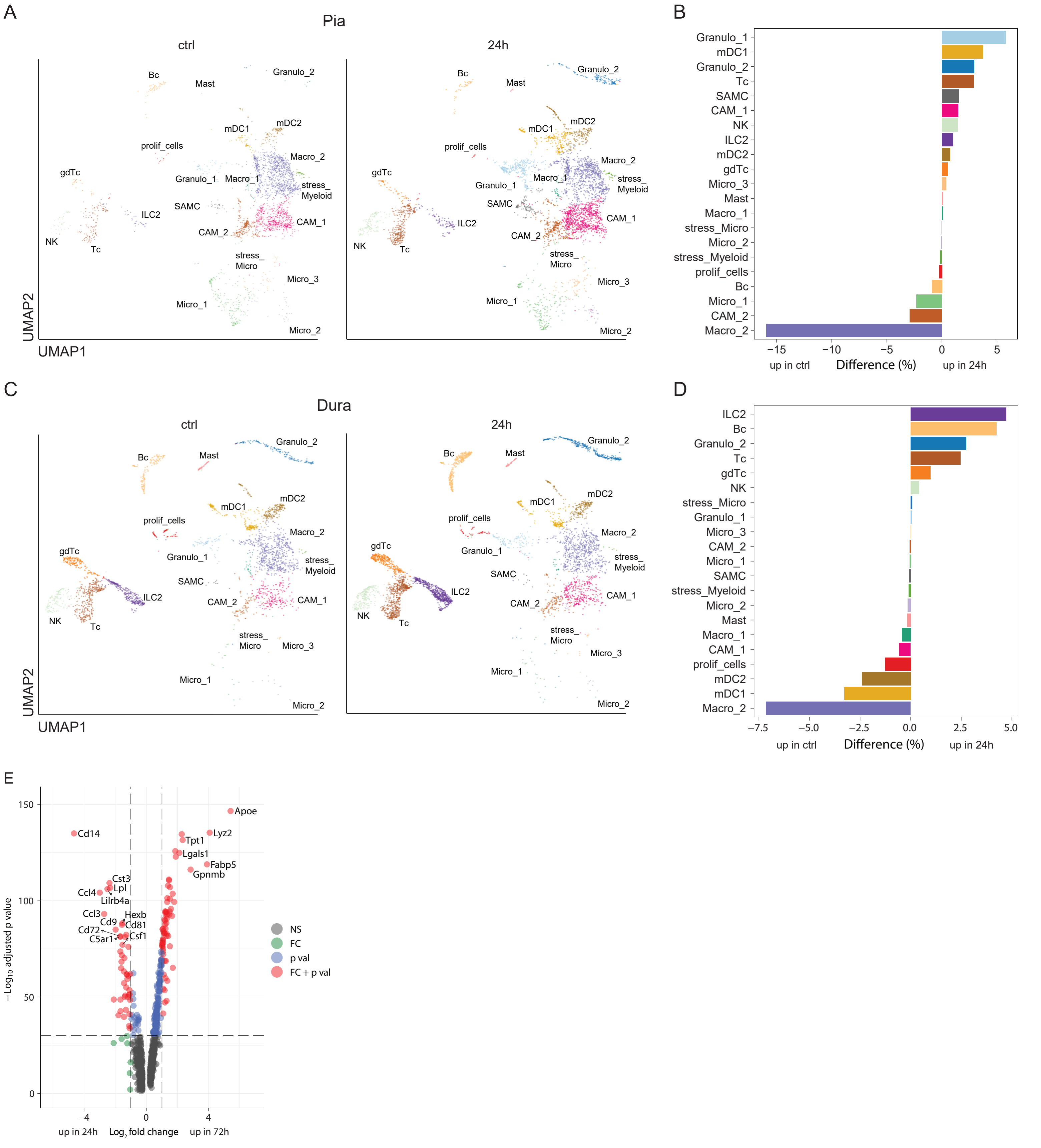

Supplementary Figure 4: scRNA-Seq identifies leukocyte compositional changes in brain associated compartments upon stroke. **(A)** UMAP plots of single cell transcriptomes obtained from Pia of sham (ctrl) and MCAO mice, 24h (24h) and 72h (72h) post stroke. **(B)** Bi-directional histograms depicting differences in relative cluster abundance between MCAO (24h and 72h post stroke) and sham (ctrl) samples as shown in (A). Differences are calculated as: percentage of all cells in the respective stroke sample minus percentage of all cells in the control sample. **(C)** UMAP plots of single cell transcriptomes obtained from Dura of sham (ctrl) and MCAO mice, 24h (24h) and 72h (72h) post stroke. **(D)** Bi-directional histograms depicting differences in relative cluster abundance between MCAO (24h and 72h post stroke) and sham (ctrl) samples as shown in (C). Differences are calculated as: percentage of all cells in the respective stroke sample minus percentage of all cells in the control sample. **(E)** Volcanoplot depicting the $\log_2$ fold change of differential expressed genes in the SAMC cluster after 24h and 72h post ischemia. Abbreviation key: Bc, B cell; CAM, central nervous system-associated macrophages; gdTc, γδ T cells; granulo, granulocytes; ILC2, innate lymphoid cells type 2; Macro, macrophage; Mast, mast cells; mDC, myeloid dendritic cell; Micro, microglia; NK, natural killer cells; proli_cells, proliferating cells; SAMC, stroke associated myeloid cells; stress_Micro, stressed Microglia; stress_Myeloid, stressed Myeloid cells; Tc, T cells. Source data for B and D are provided as a Source Data file.

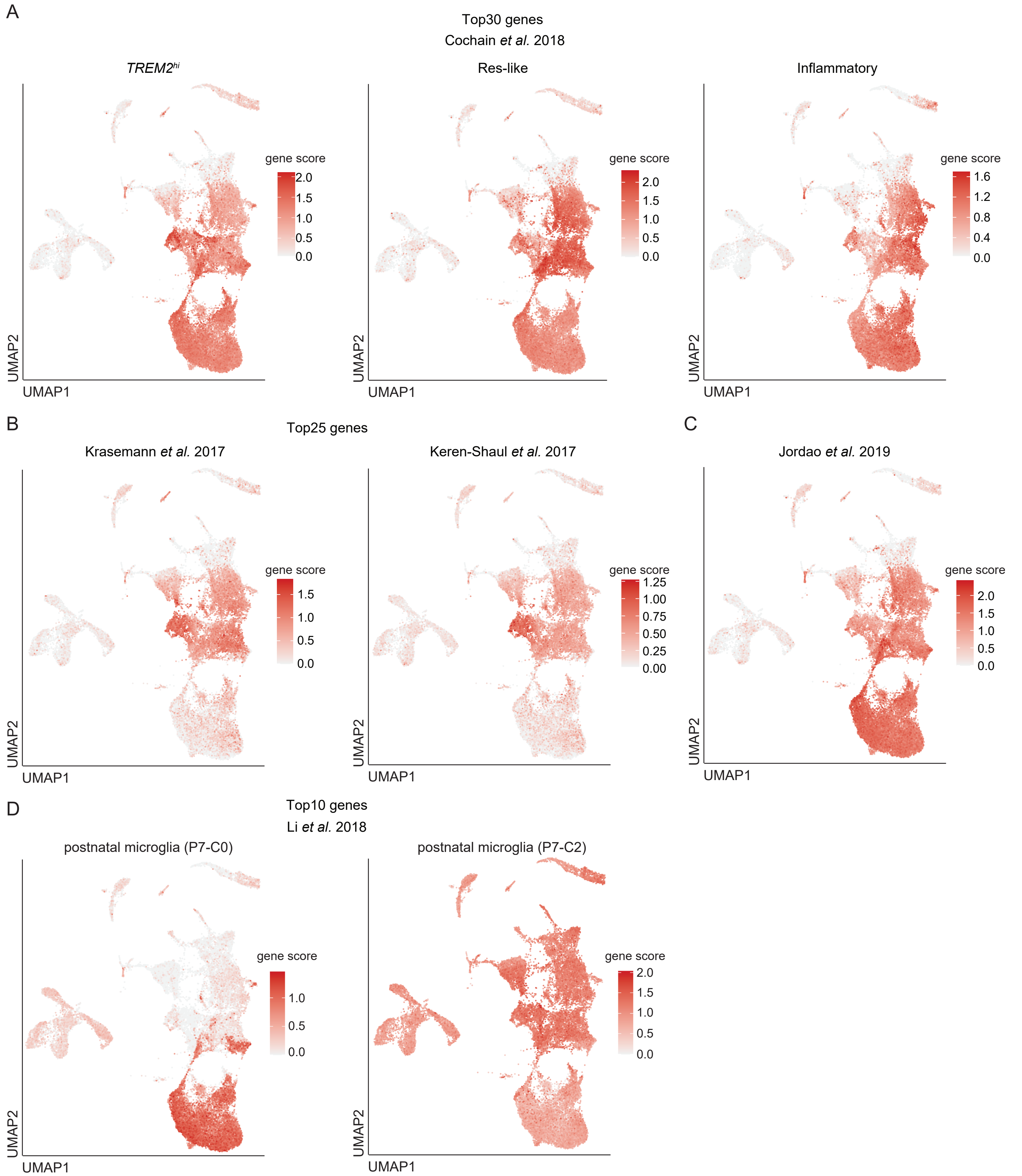

Supplementary Figure 5: Gene score feature plots of published disease-associated macrophage/microglia gene signatures. **(A)** Top30 marker genes of TREM2[hi] Macrophages, Inflammatory Macrophages and Resident-like Macrophages (IAM) from Cochain *et al.* 2018. 50 were plotted as gene score feature plots into our dataset. **(B)** Top25 marker genes of disease-associated microglia (DAM) from Keren-Shaul *et al.* 2017. 14 and Krasemann *et al.* 2017. 13 were plotted as gene score feature plots into our dataset. **(C)** Marker genes of disease-associated myeloid cells (daMC) from Jordao *et al.* 2019. 5 were plotted as gene score feature plots into our dataset. **(D)** Top25 marker genes of different postnatal day 7 microglia cells (P7-C0, P7-C2) from Li *et al.* 2019. 39 were plotted as gene score feature plots into our dataset.

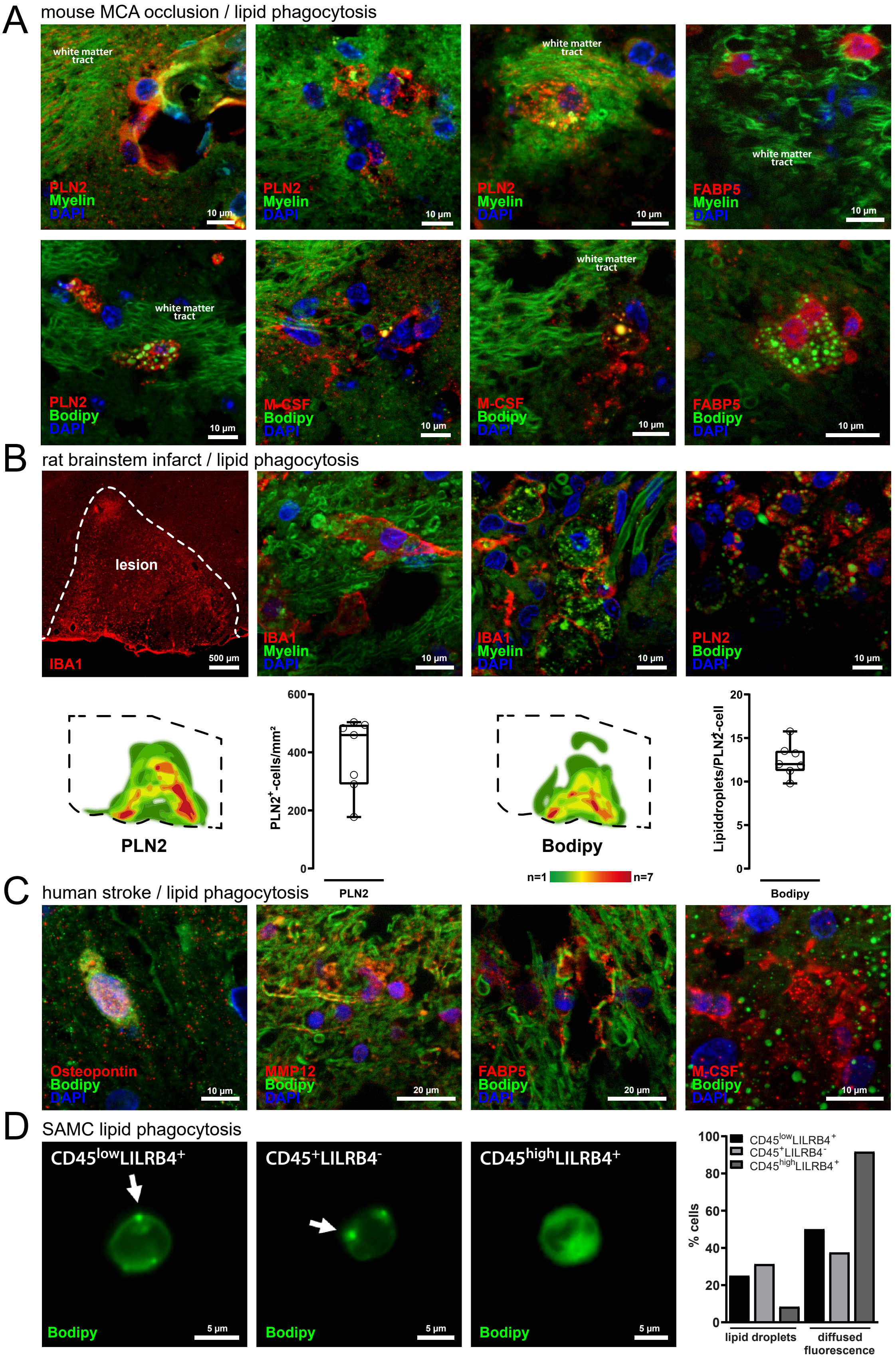

Supplementary Figure 6: Stroke-associated myeloid cells (SAMCs) demonstrate a lipid phagocytosing phenotype. **(A)** Middle cerebral artery occlusion (MCAO) was induced in mice for 45 min. Representative images of PLN2$^+$ (Perilipin-2) co-stained with FluoroMyelin, showing potential myelin phagocytosis in mice after MCAO. Representative immunofluorescence images of SAMC marker FABP5 co-stained with FluoroMyelin. Co-staining of Perilipin-2 and BODIPY reveals PLN2$^+$ intracellular vesicles enveloping BODIPY$^+$ lipid droplets. Representative immunofluorescence images of SAMC marker M-CSF and FABP5 co-stained with BODIPY (lipid droplets) within the infarcted area. **(B)** Photothrombotic brainstem ischemia was induced in wistar rats. Representative immunofluorescence images of IBA1 co-stained with FluoroMyelin and Perilipin-2 co-stained with BODIPY. The density of Perilipin-2 and Bodipy positive cells was quantified within the infarcted area (n=7)(two-sided t-test, *p<0.05). Median cell counts were presented as box plots. Lines inside the boxe denotes the median. Whiskers are: 10–90%. Heat map (drawn manually using Adobe Illustrator; Methods) depicting the distribution of BODIPY$^+$ and PLN2$^+$ cells quantified by immunohistochemistry. **(C)** Representative immunofluorescence images depicting SAMC-marker Osteopontin, MMP12, FABP5 and M-CSF co-stained with BODIPY on brain sections of stroke patients (n=5). **(D)** Bodipy staining of CD45^{low}LILRB4$^+$, CD45$^+$LILRB$^-$ and CD45^{high}LILRB4$^+$ cells after culture for 18h in the presence of 25 ug/ml ox-LDL. Images at 40x magnification. The percentage of CD45^{low}LILRB4$^+$, CD45$^+$LILRB4$^-$ and CD45^{high}LILRB4$^+$ cells presenting lipid droplets or diffused fluorescence was quantified by using ImageJ. Data are presented as mean values. Source data for B and D are provided as a Source Data file.

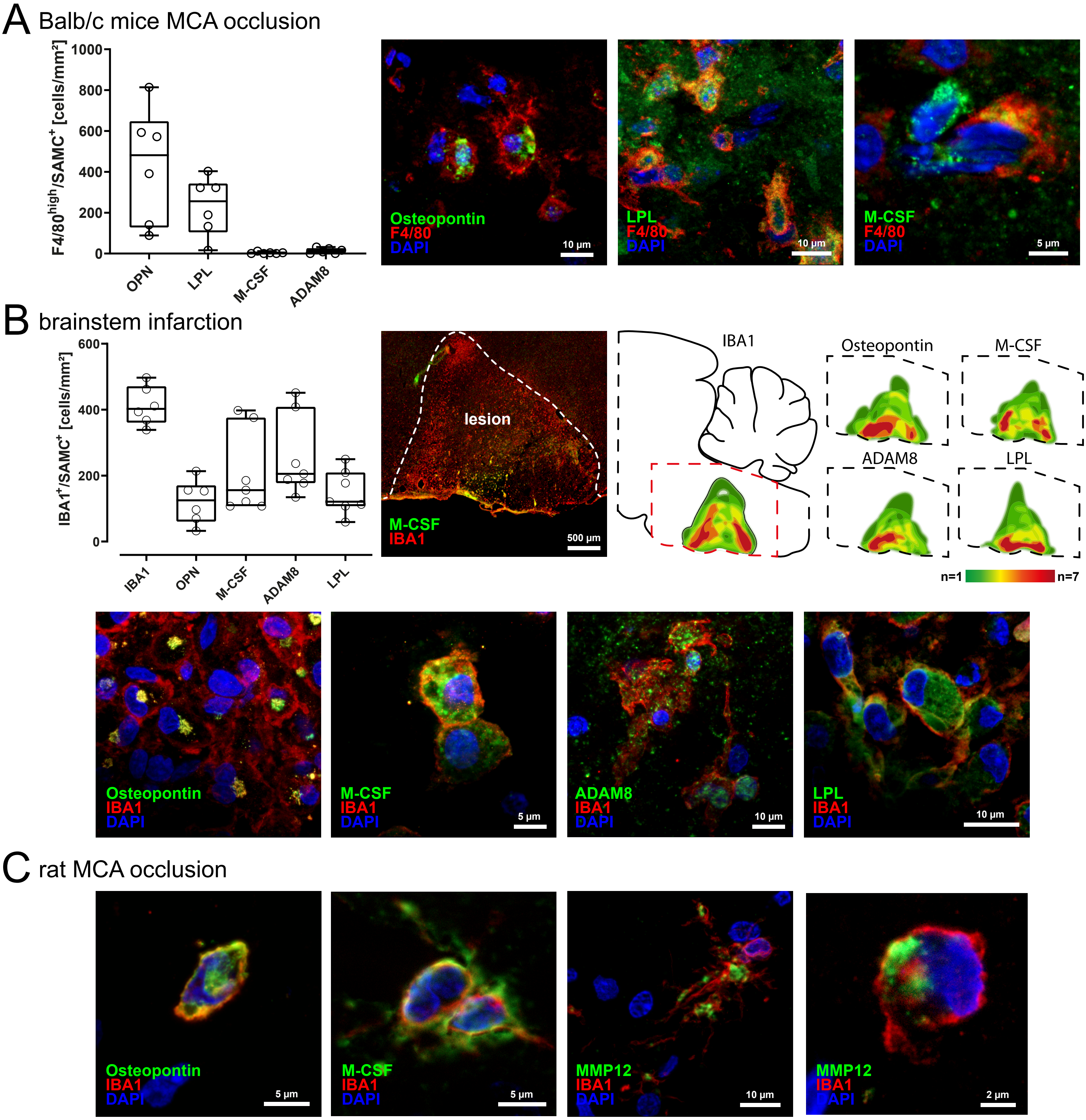

Supplementary Figure 7: Stroke-associated myeloid cells (SAMCs) are replicated in other species and stroke models. **(A)** Middle cerebral artery occlusion (MCAO) was induced in Balb/c mice for 45 min. The density of F4/80^{high}/SAMC^{+} cells was quantified within the defined sections 1-6 of 72h (n=6) post-MCAO brains. Median cell counts are presented as box plots. Lines inside the boxes denote the medians. Whiskers are: 10–90%. Coronal (approx. bregma 0-1 mm) cryosections were prepared of 72h post-MCAO brains and were co-stained for F4/80 together with Osteopontin (OPN), LPL and M-CSF. Representative Z-stacks are shown. **(B)** Photothrombotic brainstem ischemia was induced in wistar rats. The density of specific SAMC markers (Osteopontin, M-CSF, ADAM8 and LPL) together with IBA1 was quantified in post 72h animals within the infarcted area (n=7). Median cell counts are presented as box plots. Lines inside the boxes denote the medians. Whiskers are: 10–90%. Heat map (drawn manually using Adobe Illustrator; Methods) depicting the distribution of IBA1^{+}, Osteopontin^{+}, M-CSF^{+}, ADAM8^{+} and LPL^{+}-cells quantified by immunohistochemistry with color legend indicating the quantitative distribution of immune cells. Sections of infarcted brain tissue were co-stained for Iba-1 together with Osteopontin, M-CSF, ADAM8 and LPL. **(C)** MCAO was induced in wistar rats for 45 min. Sections of 72h post-MCAO brains were co-stained for IBA1 together with Osteopontin, M-CSF and MMP 12. Representative Z-stacks are shown. Source data for A and B are provided as a Source Data file.

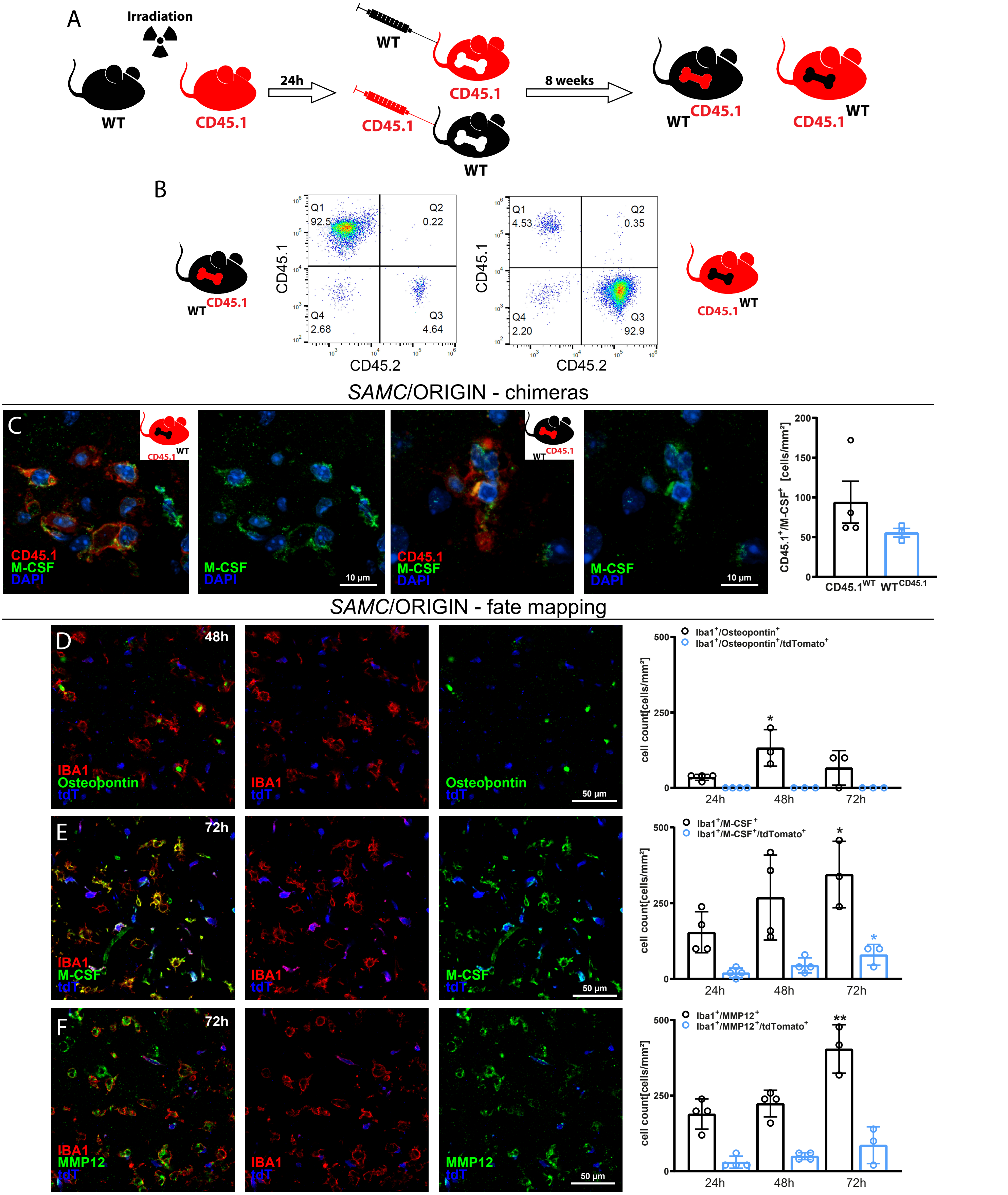

Supplementary Figure 8: Schematic representation of the chimerism approach. **(A)** Either CD45.1 or CD45.2 wildtype mice (WT) were sublethally irradiated with 7.5 Gy. After 24h, animals were reconstituted with 0.5×10^7 congenic bone marrow cells of the 'opposite' wild-type bone marrow (CD45.2 -> CD45.1 or CD45.1 -> CD45.2). **(B)** After 8 weeks of reconstitution, mice were checked for chimerism grade by flow cytometric differentiation between the allelic CD45 variants in leukocytes of tail vein blood samples. Animals with >90% chimerism were used in the experiment. **(C)** Bone marrow chimeric mice were generated with either host ($CD45.1^{WT}$, n=4) or graft ($WT^{CD45.1}$, n=3) hematopoietic cells expressing the CD45.1 congenic marker. Eight weeks after engraftment, MCAO was induced for 30 min and after 72h brain sections were co-stained for M-CSF and CD45.1. The proportion of M-CSF^{+} cells of host and graft cells was quantified within 6 areas covering the lesioned striatum. **(D-F)** MCAO was induced for 45 min in Cxcr4CreERT2 x R26CAG-LSL-tdTomato fate-reporter mice and after 24h, 48h and 72h brain sections were co-stained for tdTomato, Iba1, and (D) osteopontin, (E) M-CSF or (F) MMP12. The proportion of triple-positive (tdTomato^{+}/IBA1^{+}/SAMC^{+}) and double-positive (tdTomato^{+}/IBA1^{+}/SAMC^{+}) cells was quantified within 3-4 areas covering the lesioned striatum. Data are summed from 3 mice each and are presented as mean ± SEM (two-sided t-testt test, *p<0.05, **p<0.01). Source data for C and D are provided as a Source Data file.
