# Supplementary material for: Stroke induces disease-specific myeloid cells in the brain parenchyma and pia
Source: Nat Commun. 2022 Feb 17;13:945. doi: 10.1038/s41467-022-28593-1 (PMC8854573; doi:10.1038/s41467-022-28593-1)
Supplement: Supplementary file 3 — Description of Additional Supplementary Files [file 41467_2022_28593_MOESM3_ESM.pdf]

Description of Additional Supplementary Files

File Name: Supplementary Data 1
Description: **Technical information on scRNA-seq samples and sequencing. sum:** Summe; **mean:** Average number; **SD:** Standard deviation; **SEM:** Standard error of the mean. Column and row headers are written in bold.

File Name: Supplementary Data 2
Description: **Marker genes of Clusters in scRNA-seq dataset.** Marker genes were calculated in a one vs. all comparison. Average log FC threshold was set to 0.25, genes with an adjusted p value above 0.05 were removed. Only genes with a positive average log FC are shown. **avg_log_FC:** log fold change of the average expression between the cluster vs. all remaining clusters**; pct.1:** percentage of cells with the gene detected in the cluster; **pct.2:** percentage of cells with the gene detected in all remaining clusters; **p_val_adj:** adjusted p values (based on Wilcoxon rank sum test with Bonferroni correction). Cluster names correspond to Figure 2. Column and row headers are written in bold.

File Name: Supplementary Data 3
Description: **Differentially expressed genes in SAMC 24h vs. 72h post ischemia.** Differentially expressed genes in SAMC were calculated in a 24h vs. 72h comparison. **avg_log_FC:** log fold change of the average expression between 24h vs. 72h**; pct.1:** percentage of cells with the gene detected in the cluster; **pct.2:** percentage of cells with the gene detected in all remaining clusters; **p_val_adj:** adjusted p values (based on Wilcoxon rank sum test with Bonferroni correction). Column and row headers are written in bold.

File Name: Supplementary Data 4
Description: **Functional annotation of marker genes of the SAMC cluster.** Marker genes of cluster SAMC were analyzed by gene set enrichment analysis using Enrichr (see Methods for details). p-values were calculated using the default setting (Fisher exact test). **PPI:** protein-protein interaction **TF:** transcription factor. Column and row headers are written in bold.

File Name: Supplementary Data 5
Description: **Comparison of published datasets with SAMC.** Marker genes identified in myeloid lineage cells were extracted from different publications[5,13,14,28,30,39] and were used for comparison with the SAMC cluster. Column and row headers are written in bold.
