# Supplementary material for: Stroke induces disease-specific myeloid cells in the brain parenchyma and pia
Source: Nat Commun. 2022 Feb 17;13:945. doi: 10.1038/s41467-022-28593-1 (PMC8854573; doi:10.1038/s41467-022-28593-1)
Supplement: Supplementary file 9 — Reporting Summary [file 41467_2022_28593_MOESM9_ESM.pdf]

nature portfolio

Corresponding author(s): Prof. Dr. J. Minnerup / PD Dr. G. Meyer zu Hörste

Last updated by author(s): Nov 24, 2021

Reporting Summary

Nature Portfolio wishes to improve the reproducibility of the work that we publish. This form provides structure for consistency and transparency in reporting. For further information on Nature Portfolio policies, see our Editorial Policies and the Editorial Policy Checklist.

Statistics

For all statistical analyses, confirm that the following items are present in the figure legend, table legend, main text, or Methods section.

n/a	Confirmed	
☐	☒	The exact sample size (n) for each experimental group/condition, given as a discrete number and unit of measurement
☐	☒	A statement on whether measurements were taken from distinct samples or whether the same sample was measured repeatedly
☐	☒	The statistical test(s) used AND whether they are one- or two-sided<br>*Only common tests should be described solely by name; describe more complex techniques in the Methods section.*
☐	☒	A description of all covariates tested
☐	☒	A description of any assumptions or corrections, such as tests of normality and adjustment for multiple comparisons
☐	☒	A full description of the statistical parameters including central tendency (e.g. means) or other basic estimates (e.g. regression coefficient) AND variation (e.g. standard deviation) or associated estimates of uncertainty (e.g. confidence intervals)
☐	☒	For null hypothesis testing, the test statistic (e.g. F, t, r) with confidence intervals, effect sizes, degrees of freedom and P value noted<br>*Give P values as exact values whenever suitable.*
☒	☐	For Bayesian analysis, information on the choice of priors and Markov chain Monte Carlo settings
☒	☐	For hierarchical and complex designs, identification of the appropriate level for tests and full reporting of outcomes
☒	☐	Estimates of effect sizes (e.g. Cohen's d, Pearson's r), indicating how they were calculated

Our web collection on statistics for biologists contains articles on many of the points above.

Software and code

Policy information about availability of computer code

Data collection	Cytexpert v2.4, Kaluza (Beckman Coulter, A63881), ImageJ v1.53a (NIH).
Data analysis	FlowJo software v10.6.1 (BD), Cellranger v6, R sofware (v4.1.1 and v3.6.3), Seurat v4.0.4, scDblFinder v1.6, scMCA package, Enrichr v3.0, GraphPad Prism 5, ImageJ v1.48, Enhanced Volcano v1.5 package, Adobe Illustrator (Adobe Illustrator CS5), sctransform package (github.com/ChristophH/sctransform), sample size calculator available at http://www.stat.ubc.ca, Free-D software (v1).

For manuscripts utilizing custom algorithms or software that are central to the research but not yet described in published literature, software must be made available to editors and reviewers. We strongly encourage code deposition in a community repository (e.g. GitHub). See the Nature Portfolio guidelines for submitting code & software for further information.

Data

Policy information about availability of data

All manuscripts must include a data availability statement. This statement should provide the following information, where applicable:

- Accession codes, unique identifiers, or web links for publicly available datasets
- A description of any restrictions on data availability
- For clinical datasets or third party data, please ensure that the statement adheres to our policy

The raw scRNA-seq data of this study including cluster and sample annotations are available in the GEO repository (GSE189432). Technical details about the sequencing samples as well as the results of the differential expression data enrichment analysis are given in the method section and as supplementary data. Additional source data underlying figures 1,4,5 and supplementary figures 1,6,7 and 8 are provided in the source data file.
The processed and annotated single-cell RNA-seq dataset can be interactively explored at: https://osmzhlab.uni-muenster.de/shiny/cerebro_stroke/. The code was based on the official tutorials of the packages listed, no custom code was generated.

Field-specific reporting

Please select the one below that is the best fit for your research. If you are not sure, read the appropriate sections before making your selection.

☒ Life sciences ☐ Behavioural & social sciences ☐ Ecological, evolutionary & environmental sciences

For a reference copy of the document with all sections, see nature.com/documents/nr-reporting-summary-flat.pdf

Life sciences study design

All studies must disclose on these points even when the disclosure is negative.

Sample size

Using the sample size calculator available at http://www.stat.ubc.ca, we performed a priori sample size calculations to achieve 80% power to detect a relevant treatment effect of 25% with an alpha level of 0.05.

Data exclusions

No samples were excluded from the study. In scRNA-Seq. cell filtering was performed: Low-quality cells and cell doublets were removed by filtering cells with few genes (<200) or high number of genes (>1700-5000) for each compartment separately. Data were normalized using a recently described approach with regularized negative binomial regression called SCTransform54.
Human samples not fullfilling the diagnostic criteria were excluded from the study.

Replication

scRNA-Seq. experiments were replicated 2 times and after succesfull replication pooled for a total of n = 5 for each compartment (CNS, Dura, Pia and CP). Human histology stainings were performed on autopsies from 5 independent patients. Mouse histology stainings were performed on 6 individual mice in 3 independent experiments.
In mice, first unbiased single cell RNA-sequencing was applied to generate hypotheses. Then subsequently, core findings were replicated using multi-color flow cytometry staining for distinct immune cells, and immunohistochemistry for stroke-associated myeloid cells. All animal experiments were successfully replicated. Immunohistochemical findings were further replicated in human. For details, please refer to the methods section of the manuscript.

Randomization

Animals were allocated into experimental groups based on the treatment performed. Animals from both treatment groups are wild-type mice. Human autopsy material was chosen based on the autopsy diagnosis. Only patients with diagnosis of ischemic stroke were included into the study. Since human control groups without diagnosis of ischemic stroke were not used, random allocation is not relevant to our study.

Blinding

Data analysis of both experimental groups (animal experiments) was performed in a blinded fashion. The investigators were blinded to group allocation during data collection in experimental stroke. For human histology, no blinding was performed as only autopsies from patients with ischemic stroke were included into the study.

Reporting for specific materials, systems and methods

We require information from authors about some types of materials, experimental systems and methods used in many studies. Here, indicate whether each material, system or method listed is relevant to your study. If you are not sure if a list item applies to your research, read the appropriate section before selecting a response.

Materials & experimental systems

n/a	Involved in the study	
☐	☒	Antibodies
☒	☐	Eukaryotic cell lines
☒	☐	Palaeontology and archaeology
☐	☒	Animals and other organisms
☐	☒	Human research participants
☐	☒	Clinical data
☐	☒	Dual use research of concern

Methods

n/a	Involved in the study	
☒	☐	ChIP-seq
☐	☒	Flow cytometry
☒	☐	MRI-based neuroimaging

Antibodies

Antibodies used

anti-mouse flow cytometry antibodies:

Antigen	clone	Fluorochrome	Cat#	Manufacturer	Dilution
CD45	(30-11F)	BV510, FITC	103137, 103107	Biolegend	1:100
CD45R/B220	(RA3-6B2)	PerCP-Cy5.5	103235	Biolegend	1:100
CD3	(17A2)	Pe-Cy7	100219	Biolegend	1:200
F4/80	(BM8)	APC	123115	Biolegend	1:200
Ly-6G/Ly-6C	(RB6-8C5)	BV421	108433	Biolegend	1:200
CD11c	(N418)	AF700	117319	Biolegend	1:150
CD11b	(M1/70)	Pe	101207	Biolegend	1:800
NK-1.1	(PK136)	APC-Vio770	130-116-498	Miltenyi	1:200

anti-mouse histology antibodies: APC (1:100, raised in mouse, Merck, OP80, batch 3031622), CD3 (1:50, raised in hamster, BD Pharmingen, 550277, batch 9024853), CD45R/B220 (1:100, raised in rat, clone RA3-6B2, ThermoFisher, 14-0452-82, batch 4338655), F4/80 (1:500, raised in rat, clone CI:A3-1, Serotec/BioRad, MCA497G, batch 153472), FABP5 (1:100, raised in goat, R&D, AF1476, batch IQW022108A), Glial Fibrillary Acidic Protein (1:500, raised in mouse, clone G-A-5, Millipore, G3893, batch 029M4819V), Iba1 (1:50, raised in goat, Abcam, ab5076, batch GR3253755-2), Laminin (1:100, raised in rabbit, Abcam, ab11575, batch GR3349807-1), LPL (1:100, raised in mouse, clone LPLA4, Abcam, ab21356, batch GR3377688-1), Ly-6B.2 (1:100, raised in mouse, clone 7/4, BioRad, MCA771G, batch 1701), MAP2 (1:500, raised in chicken, Abcam, ab5392, batch GR3265288-4), M-CSF (1:100, raised in rabbit, clone ERP20948, Abcam, ab233387, batch GR3247126-2), MMP12 (1:100, raised in rabbit, Abcam, ab128030), MS2/Adam8 (1:100, raised in rabbit, clone EPR22688-44, Abcam, ab255608, batch GR3287934-2), NeuN (1:150, raised in rabbit, clone 27-4, Millipore, MABN140, batch 3230506), Osteopontin (1:100, raised in rabbit, Abcam, ab63856, batch GR3300805-2), Perilipin-2 (1:100, raised in rabbit, Novusbio, NBP2-48532, batch 572), TSpan4 (1:100, raised in rabbit, LSBio, LS-B11508, batch 104449), IdU (1:200, raised in mouse, clone B44, BD Pharmingen, BD347580, batch 5295722), Fluoromyelin Green (1:100, ThermoFisher, F34651), Streptavidin-HRP (1:100, Biolegend, 405210, batch B293545), Bodypi 493/503 (1:1000, Molecular Probes, D3922, batch 2256834), anti-rat IgG Biotin (1:100, raised in goat, Abcam, ab6844), Alexa Fluor 488 anti-rat IgG (1:100, raised in goat, Life, A-11006), Alexa Fluor 488 anti-rabbit IgG (1:100, raised in goat, Life, A-11008), Alexa Fluor 488 anti-hamster IgG (1:100, raised in goat, Life, A21110), Alexa Fluor 488 Streptavidin (1:100, Life, S-11223, batch 1990311), Alexa Fluor 488 anti-mouse IgG (1:100, raised in goat, Life, A-11001), Alexa Fluor 594 anti-rabbit IgG (1:100, raised in donkey, Life, A-21207), Alexa Fluor 594 anti-goat IgG (1:100, raised in goat, Dianova, 705-585-003), Alexa Fluor 594 anti-mouse IgG (1:100, raised in goat, Life, A-11005), Alexa Fluor 594 anti-rat IgG (1:100, raised in goat, Life, A-11007), Alexa Fluor 594 anti-hamster IgG (1:100, raised in goat, Life, A-21113), Alexa Fluor 594 Streptavidin (1:100, Life, S-11227, batch 1991448), Fluoromount with DAPI (Life, 00-4952-52, batch E136821), Click-iT Plus TUNEL Assay (ThermoFisher, C10617, batch 2151745)

anti-human histology antibodies: anti-Osteopontin (1:100, raised in rabbit, Abcam, ab63856, batch GR3300805-2), anti-LPL (1:100, raised in mouse, clone LPLA4, Abcam, ab21356, batch GR3377688-1), anti-CSF1/M-CSF (1:100, raised in rabbit, clone ERP20948, Abcam, ab233387, batch GR3247126-2), anti-MS2/Adam8 (1:100, raised in rabbit, clone EPR22688-44, Abcam, ab255608, batch GR3287934-2), anti-MMP12 (1:100, Abcam), anti-MAP2 (1:500, raised in rabbit, Abcam, ab128030), anti-FABP5 (1:25, raised in goat, R&D, AF1476, batch IQW022108A) and anti-IBA1 (1:50, raised in goat, Abcam, ab5076, batch GR3253755-2)

Validation

All antibodies used are commercially available and were validated by the manufacturer for the method they were used for.

Biolegend: "Each lot of this antibody is quality control tested by immunofluorescent staining with flow cytometric analysis. For flow cytometric staining, the suggested use of this reagent is ≤ 0.25 µg per 10^6 cells in 100 µl volume. It is recommended that the reagent be titrated for optimal performance for each application." Exact description for every antibody on the manufacturer's website under the respective catalog number.

Invitrogen: "This xxx antibody has been tested by flow cytometric analysis as a second step to detect mouse xxx primary antibodies. This can be used at less than or equal to 0.125 µg per test. A test is defined as the amount (µg) of antibody that will stain a cell sample in a final volume of 100 µL. Cell number should be determined empirically but can range from 10^5 to 10^8 cells/test. It is recommended that the antibody be carefully titrated for optimal performance in the assay of interest." Exact description for every antibody on the manufacturer's website under the respective catalog number.

Miltenyi: "QC tested" for more Information on testing procedure go to "https://www.miltenyibiotec.com/DE-en/products/macs-antibodies/Antibody-production-development-and-quality-control.html" "In order to compare the epitope specificity of an antibody, the clone being used is compared with other known clones recognizing the same antigen in a competition assay. Cells were incubated with an excess of purified unconjugated antibody followed by staining with fluorochrome-conjugated antibodies of other known clones against the same marker. Based on the fluorescence signal obtained, the clones were identified as recognizing completely overlapping (++), partially overlapping (+), or completely different epitopes (-) of the marker. Selected fluorochrome conjugated antibodies from Miltenyi Biotec were compared to commercially available hybridoma clones in flow cytometry analysis."

BD Biosciences: "All flow cytometry reagents are titrated on the relevant positive or negative cells. Quality control testing of new, manufacutred lots are performed side-by-side with a previously accepted lot as a control, helping to serve as a reference for comparison and assuring that performance of the new lot is both reliable and consistent."

R&D Systems: "R&D Systems manufactures the majority of our products offered and as a result, these products are tested and validated at our facilities. R&D Systems carefully tests every antibody we produce to ensure outstanding performance. Our commitment to quality allows you to be confident in your results and help you generate publication-quality data. Here's how:
Our rigorous testing includes the use of internally generated tissue microarrays comprised of normal and pathological tissues from multiple donors. Even though our antibodies are for research use only, we use the same instrumentation that is used in IHC diagnostic labs, including automated slide staining system and a high-throughput slide scanner for collecting and archiving high-resolution images. Our IHC protocols are standardized and based on approved SOPs to assure consistency and reproducibility of generated data. When possible, we do side-by-side testing of our antibodies using both chromogenic and fluorescence detection techniques."

Abcam: "Our Abpromise guarantee covers product applications & species that have been tested in our own labs, by our suppliers, or by selected trusted collaborators."
Immunohistochemistry and immunocytochemistry: "IHC and ICC determine whether an antibody recognizes the correct protein based on cellular and subcellular localization. Antibody specificity is confirmed by looking at cells that either do or do not express the target protein within the same tissue. Initially, our scientists will review the available literature to determine the best cell lines and tissues to use for validation. We then check the protein expression by IHC/ICC to see if it has the expected cellular localization (Figure 3). If the localization of the signal is as expected, this antibody will pass and is considered suitable for use in IHC/ICC.
We use a variety of methods, including staining multi-normal human tissue microarrays (TMAs), multi-tumor human TMAs, and rat or mouse TMAs during antibody development. These high-throughput arrays allow us to check many tissues at the same time, providing uniformly as all tissues are exposed to the exact same conditions. "
Flow Cytometry: "When validating antibodies for flow cytometry, we are careful to optimize every step of the staining protocol, including fixation, permeabilization, and washing. To do this, our scientists review the available literature to understand which cell types and conditions are best suited to validate specific antibodies.

We include relevant controls, routinely running unstained, positive, negative, isotype, viability, Fc-blocking, fluorescence minus one (FMO), and single-staining controls. For an FMO control, we stain all our samples with fluorescent conjugates except the one that is being tested. This shows the contribution of the other fluorescent conjugates in the signal of the unlabeled channel. This control is important for determining non-specific binding of an antibody.
Isotype controls are a good negative control that allows us to determine background signal from the signal given by specific antibody binding. These controls use primary antibodies matching the isotype of the primary antibody you are validating but which do not have specificity for the target. We also use many KO cell lines in our flow cytometry validation whenever these are available. If an antibody gives a positive flow cytometry signal and passes all of our control experiements we will make this antibody available for purchase and state that it is suitable for use in flow cytometry."

Merck/Millipore: "To support our multi-step, multi-application validation process, we have a tissue and blot library with over 1300 lysates, allowing us to precisely determine each antibody's specificity. At Merck Millipore, we have the advantage of having an entire cell analysis technology development team in-house. We validate antibodies for flow cytometry using our own guava easyCyte™ dual-laser microcapillary instruments. Similarly, our in-house bead-based immunodetection team helps us validate antibodies using the trusted Luminex xMAP platform. Using confocal microscopes and high-throughput IHC instruments, we can obtain accurate data faster than manual imaging. Further scientific review determines whether staining patterns conform to published subcellular expression. For immunohistochemistry, we include negative controls, to confirm the signal."

BioRad: "Antibodies are evaluated against multiple biologically relevant cell lysates and tissues that express the endogenous target. Where relevant, negative controls and other important samples, such as human serum and human or rodent tissue lysates, are included. For each antibody, we carefully review the literature to identify lysates expected to express the target and use this to evaluate the antibody."

ThermoFisher/Invitrogen: "Our antibodies are being tested using at least 1 of the following methods to ensure proper functionality in researcher's experiments:
Knockout—expression testing using CRISPR-Cas9 cell models
Knockdown—expression testing using RNAi to knockdown gene of interest
Independent antibody verification (IAV)—measurement of target expression is performed using two differentially raised antibodies recognizing the same protein target
Cell treatment—detecting downstream events following cell treatment
Relative expression—using naturally occurring variable expression to confirm specificity
Neutralization—functional blocking of protein activity by antibody binding
Peptide array—using arrays to test reactivity against known protein modifications
SNAP-ChIP™—using SNAP ChIP to test reactivity against known protein modifications
Immunoprecipitation-Mass Spectrometry (IP-MS)—testing using immunoprecipitation followed by mass spectrometry to identify antibody targets"

Novus biologicals: "Novus guarantees that every product we sell will work in the application and species listed on our website and datasheets." Used are different validation methods:
"Genetic strategic validation: Expression of the target protein is compared before and after knockout or knockdown using CRISPR/CAS9 or siRNA/shRNA. If protein expression following knockout or knockdown is substantially reduced, then antibody specificity is ensured.
Orthogonal validation: The target protein is examined with an antibody independent strategy and compared with results from an antibody-dependent strategy. A correlation between these two strategies indicates specificity between the antibody and its target protein. Examples of antibody independent techniques may include in situ hybridization, quantitative PCR, RNA-seq or mass spectrometry.
Independent Antibody Validation: The data generated using several antibodies (ideally targeting different epitopes) in the same protein is compared (e.g. molecular weight and cellular localization). Consistent results imply antibody selectivity to the target protein.
Expression of Tagged Proteins Validation: A tagged protein is used as a standard for comparison in Western blotting and/or immunocytochemistry (ICC). For example, if the distribution of the tagged protein overlaps with the immunofluorescence signal, then antibody specificity is confirmed.
Biological Strategies Validation: These strategies use defined biological or chemical modulation of protein expression to demonstrate antibody specificity to the target protein. The data is compared across multiple cell lines including positive and negative expressing cells, and multiple species, if applicable."

Animals and other organisms

Policy information about studies involving animals; ARRIVE guidelines recommended for reporting animal research

Laboratory animals

C57BL/6J mice (WT, adult, male, 10-16 weeks of age), B6.SJL-PtprcaPepcb/BoyJ mice (i.e. CD45.1 congenic C57BL/6, adult, male, 10-16 weeks of age), and adult male Wistar rats adult, male, 10-16 weeks of age) were purchased from Charles River. Balb/c male mice (adult, male, 10 weeks of age) were purchased from Istanbul Medipol University Animal Research laboratory (MEDITAM). Cxcr4CreER/WtR26CAG-LSL-tdT mice (adult, male, n=24) were bred in the central animal facility of Institute of Pharmacology and Toxicology, Jena University Hospital, Jena, Germany. Animals were kept in standard housing conditions with 12h dark/light cycle, ambient temperature between 20-24°C, humidity between 45%-65% and food and water ad libitum.

Wild animals

The study did not involve wild animals.

Field-collected samples

The study did not involve field-collected samples.

Ethics oversight

All animal experiments (C57BL/6J mice, B6.SJL-PtprcaPepcb/BoyJ mice and Wistar rats) were approved by the responsible state authority (LANUV NRW and Regierungspraesidium Oberbayern) and the ethics committees of the Westfälische Wilhelms-Universität Münster and University Hospital München under reference number 81-02.04.2018.A316 and were performed in accordance to local regulations. The experiments with Balb/c mice were carried out in accordance with National Institutes of Health (NIH) guidelines for

the care and use of laboratory animals and approved by local government authorities (Istanbul Medipol University Animal Research Ethical Committee, under reference number: E-38828770-772.02-3878; 19.08.2021/57). Cxcr4CreER/WtR26CAG-LSL-tdT mice were approved by the local governmental authorities (Landesamt für Verbraucherschutz, Thüringen, Germany) under the approval reference number 02-075/16.

Note that full information on the approval of the study protocol must also be provided in the manuscript.

Human research participants

Policy information about studies involving human research participants

Population characteristics	Gender, Age, Autopsy diagnosis:<br>female, 86 years, left middle cerebral artery territory infarct<br>male, 55 years, left middle cerebral artery territory infarct<br>male, 67 years, left middle cerebral artery territory infarct<br>female, 84 years, left middle cerebral artery territory infarct<br>female, 50 years, right middle cerebral artery territory infarct
Recruitment	Autopsies from patients with ischemic stroke were included in the study. Patients have been recruited by agreement of legal representatives. Hence, selection bias cannot be ruled out.
Ethics oversight	All patients or legal representatives provided written informed consent for scientific use. The study was performed in accordance with the declaration of Helsinki and approved by the local ethics committees of the Ärztekammer Westfalen-Lippe and Westfälische Wilhelms-University, under reference number 2017-210-f-S.

Note that full information on the approval of the study protocol must also be provided in the manuscript.

Clinical data

Policy information about clinical studies

All manuscripts should comply with the ICMJE guidelines for publication of clinical research and a completed CONSORT checklist must be included with all submissions.

Clinical trial registration	no clinical trial
Study protocol	no clinical trial
Data collection	no clinical trial
Outcomes	no clinical trial

Dual use research of concern

Policy information about dual use research of concern

Hazards

Could the accidental, deliberate or reckless misuse of agents or technologies generated in the work, or the application of information presented in the manuscript, pose a threat to:

No	Yes	
☒	☐	Public health
☒	☐	National security
☒	☐	Crops and/or livestock
☒	☐	Ecosystems
☒	☐	Any other significant area

Experiments of concern

Does the work involve any of these experiments of concern:

No	Yes	
☒	☐	Demonstrate how to render a vaccine ineffective
☒	☐	Confer resistance to therapeutically useful antibiotics or antiviral agents
☒	☐	Enhance the virulence of a pathogen or render a nonpathogen virulent
☒	☐	Increase transmissibility of a pathogen
☒	☐	Alter the host range of a pathogen
☒	☐	Enable evasion of diagnostic/detection modalities
☒	☐	Enable the weaponization of a biological agent or toxin
☒	☐	Any other potentially harmful combination of experiments and agents

Flow Cytometry

Plots

Confirm that:

☒ The axis labels state the marker and fluorochrome used (e.g. CD4-FITC).

☒ The axis scales are clearly visible. Include numbers along axes only for bottom left plot of group (a 'group' is an analysis of identical markers).

☒ All plots are contour plots with outliers or pseudocolor plots.

☒ A numerical value for number of cells or percentage (with statistics) is provided.

Methodology

Sample preparation	All mouse cells were isolated and processed for flow cytometry as described in the materials and methods section.
Instrument	BD FACS Aria III, Beckman Coulter Gallios, Beckman Coulter Cytoflex S
Software	FACS Diva v8, Kaluza , FlowJo v10.6.1, CytExpert v2.4
Cell population abundance	Cell population abdundances, post-sort fractions and how they were determined are described in detail within the manuscript or the materials and methods section.
Gating strategy	Detailed gating strategies for all flow cytometry and cell sorting experiments are included in the supplementary figures. In general, depris was excluded by gating on Leukocytes in the FSC/SSC plot. Next it was gated on single cells using FSC-H/FSC-W or FSC-A/FSC-H and SSC-H/SSC-W or SSC-A/SSC-H and afterwards dead cells were excluded by using a live/dead marker. In some experiments, it was then gated on tissue resident leukocytes by gating on CD45iv-CD45+ cells.

☒ Tick this box to confirm that a figure exemplifying the gating strategy is provided in the Supplementary Information.
